# Genotypic‐Phenotypic Correlations in Non‐HbE β‐Thalassemia Intermedia: A Geographic Systematic Review

**DOI:** 10.1002/mgg3.70330

**Published:** 2026-11-02

**Authors:** Kavindya Fernando, Shiromi Perera, Anuja Premawardhena

**Affiliations:** ^1^ Department of Biochemistry and Clinical Chemistry, Faculty of Medicine University of Kelaniya Ragama Sri Lanka; ^2^ NK Ceylon Institute of Medical Research Negombo Sri Lanka; ^3^ Department of Medicine, Faculty of Medicine University of Kelaniya Ragama Sri Lanka; ^4^ University Medical Unit Colombo North Teaching Hospital Ragama Sri Lanka

**Keywords:** β‐Thalassemia intermedia, genetic modifiers, genotypic diversity, phenotypic correlation, population genetics

## Abstract

**Background:**

β‐thalassemia intermedia (β‐TI), now more commonly classified within the spectrum of non‐transfusion‐dependent thalassemia (NTDT), is a clinically heterogeneous disorder in which disease severity is determined not only by β‐globin mutations but also by co‐inherited genetic modifiers that vary across populations. These include α‐globin gene alterations, fetal haemoglobin (HbF)‐inducing polymorphisms, and other regulatory loci that influence globin chain balance.

**Methods:**

A systematic review was conducted in accordance with PRISMA 2020 guidelines. PubMed was searched from inception to May 2026, supplemented by citation tracking and manual screening. Studies reporting genotype–phenotype correlations in β‐TI were included. Data were synthesized qualitatively by geographic region.

**Results:**

Twenty‐one studies were included. The available evidence suggests Mediterranean populations are primarily influenced by mild β‐globin mutations. In Middle Eastern populations, HbF‐inducing polymorphisms, particularly the XmnI variant, played a prominent role, often mitigating severe genotypes. Among the included studies, Asian populations exhibited the greatest molecular heterogeneity, with α‐globin gene dosage alterations, including triplication, contributing substantially to disease expression.

**Conclusion:**

The available evidence suggests regional differences in the contribution of genetic modifiers to the β‐thalassemia intermedia phenotype. Recognition of these patterns may improve understanding of genotype–phenotype variability and may help inform future approaches to diagnosis, prognostication, and individualized therapeutic strategies.

## Graphical abstract

β‐Thalassemia intermedia exhibits distinct region‐specific molecular patterns shaped by three interacting genetic axes: β‐globin mutation severity, HbF‐inducing modifiers, and α‐globin gene dosage. Mediterranean populations are predominantly mutation‐driven, Middle Eastern populations rely on HbF compensation, and Asian populations are governed by globin chain imbalance, findings with direct implications for precision diagnosis and therapy.

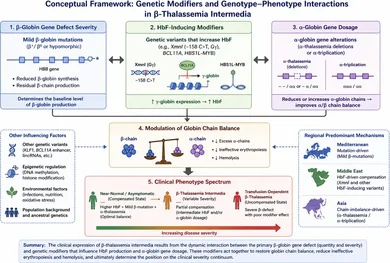

## 1 Introduction

β‐thalassemia intermedia (β‐TI) represents a heterogeneous clinical spectrum between asymptomatic carrier states and transfusion‐dependent β‐thalassemia major. Patients may present in later childhood or adulthood with varying degrees of anemia, ranging from incidental detection to clinically significant disease requiring intermittent or regular transfusion (Cao and Galanello 2010; D. Weatherall and Clegg 1981). Historically, the term β‐TI has been used to describe patients with an intermediate clinical phenotype. Although current guidelines increasingly favor the term non‐transfusion‐dependent thalassemia (NTDT), we have retained the historical terminology throughout this review because it was consistently used in the included studies and best reflects the available literature.

The clinical phenotype of β‐TI is not determined solely by the β‐globin genotype but is influenced by a complex interplay of genetic modifiers that regulate globin chain balance and erythropoiesis (Jaing et al. 2021; Mettananda 2015; Thein 2005, 2018). These include the severity of β‐globin mutations, co‐inheritance of α‐thalassemia, α‐globin gene triplication, and polymorphisms in the γ‐globin gene expression (Figure 1).

**FIGURE 1:**
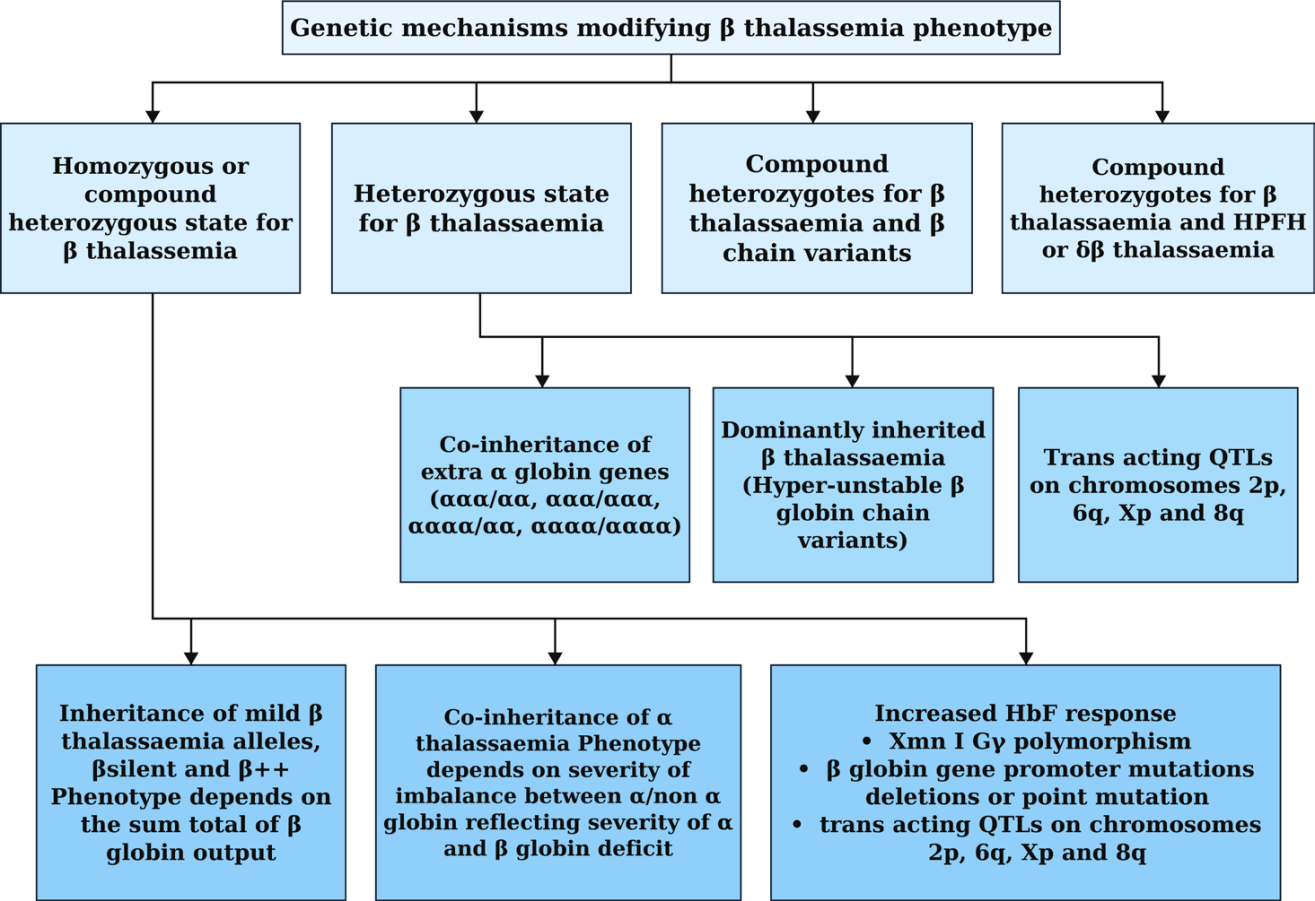
Genetic mechanisms underlying β‐thalassemia intermedia phenotype. The diagram illustrates how different genetic modifiers, including mild β‐thalassemia alleles, α‐thalassemia co‐inheritance, and trans‐acting QTLs, contribute to the wide phenotypic spectrum.

Co‐inheritance of α‐thalassemia reduces excess α‐globin chain production, thereby ameliorating globin chain imbalance and severity. In contrast, α‐globin gene triplication exacerbates α‐chain excess and may convert otherwise asymptomatic heterozygous β‐thalassemia into clinically significant disease.

In addition, HbF‐modulating genetic factors play a crucial role in phenotypic variability. Polymorphisms in the γ‐globin gene promoter, such as the XmnI site, and trans‐acting quantitative trait loci (QTLs), including BCL11A and the HBS1L‐MYB intergenic region, are key regulators of fetal haemoglobin production (Lettre et al. 2008; Sankaran et al. 2008). These factors can substantially increase HbF levels, partially compensating for β‐globin deficiency and mitigating disease severity. Other genetic conditions, such as δβ‐thalassemia and hereditary persistence of fetal haemoglobin (HPFH), further contribute to this spectrum of clinical variability.

The relative contribution of these mechanisms varies significantly across geographic and ethnic populations. Such variation is likely shaped by historical migration, founder effects, genetic drift, and selective pressure from malaria (Weatherall and Clegg 2001). Understanding these regional patterns is increasingly important in clinical practice, particularly as migration has increased the diversity of thalassemia genotypes encountered worldwide.

This systematic review aimed to synthesize genotype–phenotype correlations in non‐HbE β‐thalassemia intermedia across geographic regions. Specifically, we examined regional differences in β‐globin mutation patterns, co‐inherited genetic modifiers, and associated clinical phenotypes and propose an integrated framework for understanding population‐specific disease expression.

## 2 Methods

### 2.1 Study Design and Reporting Standards

This systematic review was conducted and reported in accordance with the PRISMA 2020 (Preferred Reporting Items for Systematic Reviews and Meta‐Analyses) guidelines (Page et al. 2021).

### 2.2 Search Strategy

A systematic literature search was conducted using PubMed from database inception to May 2026. The search strategy combined Medical Subject Headings (MeSH) and free‐text terms related to β‐thalassemia intermedia, genotype, phenotype, and genetic modifiers, and clinical severity. Boolean operators (AND, OR) were used to combine search terms. The complete PubMed search strategy, including all search terms and Boolean operators, is provided in Table S1.

To enhance the completeness of the search, additional studies were identified through manual screening of reference lists of relevant articles, citation tracking, and the “similar articles” feature in PubMed. In addition, Google Scholar was searched to identify potentially relevant studies not captured in the primary database. Only articles published in English were included.

### 2.3 Eligibility Criteria

Studies were included if they met the following criteria: original research involving human participants with β‐thalassemia intermedia, reporting genotype–phenotype correlations and/or genetic modifiers (such as α‐globin gene variations or HbF‐related polymorphisms). Eligible study designs included observational studies such as cohort, case–control, cross‐sectional, prospective, and descriptive studies conducted across any geographic region.

Studies were excluded if they were case reports or small case series, review articles, editorials, or conference abstracts without full data. Animal or in vitro studies, studies lacking relevant genotype or phenotypic information, non‐English publications, or studies focused primarily on HbE β‐thalassemia.

### 2.4 Study Selection

All records were managed using a structured spreadsheet in Microsoft Excel, and duplicate entries were removed prior to screening. Two reviewers (SP and KF) independently screened titles and abstracts for eligibility. Full‐text articles were subsequently assessed based on predefined inclusion and exclusion criteria. Disagreements between reviewers were resolved through discussion and consensus, with arbitration by a third reviewer (AP) when necessary.

The study selection process is shown in Figure 2. The search identified 2166 records. After removal of 801 duplicates, 1365 records were screened by title and abstract. A total of 137 full‐text articles were assessed for eligibility, of which 116 were excluded because they did not involve β‐thalassemia intermedia populations, lacked genotype–phenotype data, were review or editorial articles, or focused on HbE β‐thalassemia. Twenty‐one studies were included in the final qualitative synthesis. Detailed reasons for full‐text exclusion are provided in Table S2.

**FIGURE 2:**
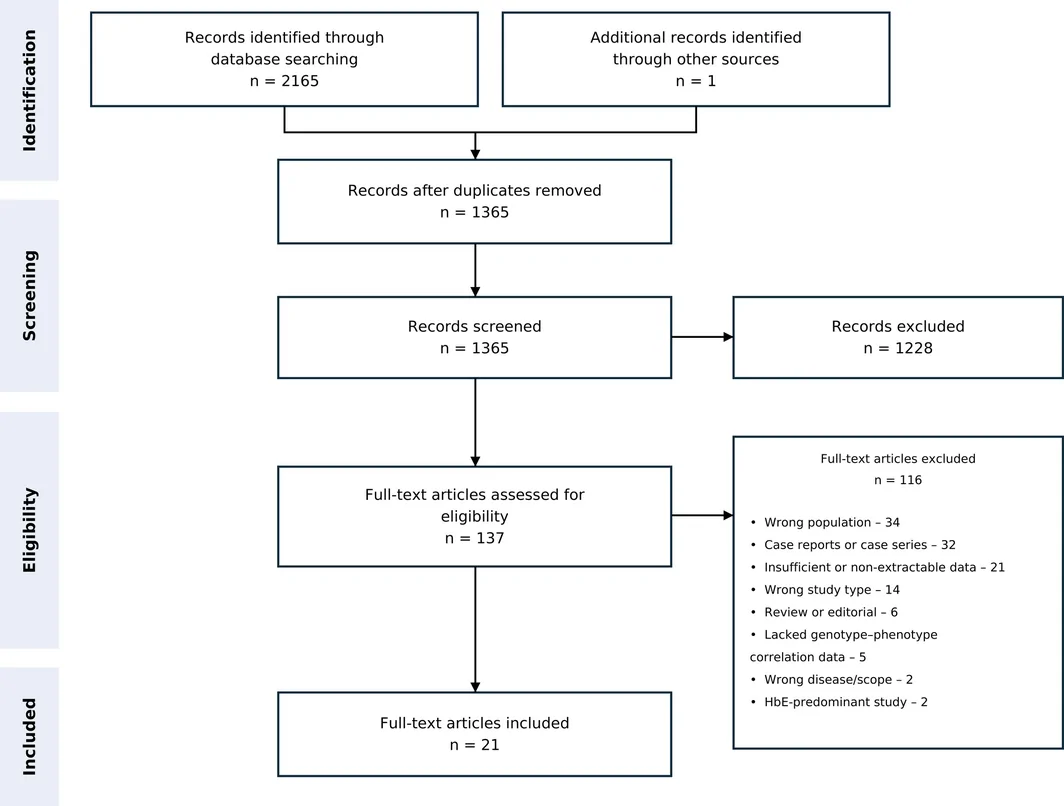
PRISMA flow diagram of study selection.

### 2.5 Data Extraction

Data extraction was performed independently by two reviewers using a standardized data collection form. The extracted variables included country, geographic region, study design, sample size, population characteristics, and genetic and clinical parameters. These comprised genotype information, including β‐globin mutations and co‐inherited modifiers, as well as key genetic modifiers such as α‐globin gene variations and XmnI polymorphism. Phenotypic characteristics, including transfusion dependence, haemoglobin levels, and clinical severity, were also recorded, along with the main findings of each study. Any discrepancies between reviewers were resolved through discussion and consensus. The completed data extraction sheet is provided in Table S3.

### 2.6 Phenotype Harmonization

Phenotypic data were harmonized where possible into transfusion‐dependent thalassemia (TDT), non‐transfusion‐dependent thalassemia (NTDT), and no regular transfusion requirement, based on reported transfusion status (Taher et al. 2018). Where studies used non‐standard categories such as mild, moderate, or severe disease, these classifications were retained as originally reported and interpreted cautiously. A study‐level crosswalk documenting the original phenotype definitions, harmonized classifications, and associated assumptions or limitations is provided in Table S5.

### 2.7 Quality Assessment

The methodological quality of included observational studies was assessed using an adapted Newcastle–Ottawa Scale (NOS) (Wells et al. 2014). Studies were evaluated across domains of participant selection, comparability, genetic characterization, and clinical phenotype reporting. Due to substantial heterogeneity in study design and reporting, quality assessment was used to support interpretation of findings rather than as a basis for study exclusion. The detailed assessment is presented in Table S4.

Overall, the included studies were of moderate to high methodological quality. However, several methodological limitations were identified. Most studies were conducted at single centers with relatively small sample sizes, limiting the generalizability of their findings. The scope of genetic modifier analysis varied considerably, with earlier studies often evaluating only classical modifiers such as α‐thalassemia and the XmnI polymorphism, whereas more recent studies incorporated additional regulatory loci. Furthermore, phenotype definitions were not uniform across studies, with inconsistent classification of transfusion dependence and disease severity. Molecular diagnostic techniques also differed according to the study period and available technology. These methodological differences were considered during data synthesis, and regional comparisons were interpreted cautiously.

### 2.8 Data Synthesis

A qualitative synthesis was performed because of heterogeneity in study design, genetic variables, and outcome definitions. Findings were grouped according to geographic region: Mediterranean, Middle Eastern, European, and Asian populations. Regional patterns in genotype–phenotype relationships were summarized narratively and compared using structured tables and a qualitative evidence map of dominant classifying the relative contribution of key genetic modifiers within each region into four tiers (not reported/rare, minor, prominent, predominant), assigned independently by two reviewers based on the consistency and magnitude with which each modifier was reported across studies within that region, with disagreements resolved by discussion.

## 3 Results

### 3.1 Study Selection

The search identified 2166 records. After removal of 801 duplicates, 1365 records were screened by title and abstract. A total of 137 full‐text articles were assessed for eligibility, and 21 studies were included in the final qualitative synthesis. The characteristics of included studies and regional genotype–phenotype patterns are summarized in Table 1.

**TABLE 1:** Regional distribution of genotype–phenotype patterns and genetic modifiers in β‐thalassemia intermedia. Data are synthesized from multiple observational studies across different geographic regions.

Region	Country	Study sample (*n*)	Genotype pattern	Key modifiers	Predominant mutations	Clinical phenotype	References
Mediterranean	Cyprus	27	All are homozygous β‐TI	α thalassemia 52% (14/27)	IVS‐I‐110 (G>A) (β^+^) 80% IVS‐I‐6 (T>C) (mild β^+^) 12% IVS‐I‐1 (G > A) (β^0^) 5% (Kountouris et al. 2016)^a^	NTDT—81% (22/27) TDT—18% (5/27)	(Wainscoat et al. 1983)
	Sardinia	61	72.1% (44/61) homozygous and 27.9% (17/61) compound heterozygous β‐TI	α thalassemia 48% (29/61) Xmn I Gγ +/− 28% (17/61)	CD 39 (C>T) (β^0^) 84% CD 6 (−A) (β^0^) 16%	All NTDT	(Galanello et al. 1989)
	Italy	165	90% (149/165) Homozygous or compound heterozygous β‐TI 10% (16/165) Heterozygous β‐TI	Mild β alleles (β^+^/β^+^or β^+^/β^0^) 68% (101/149) Xmn I Gγ +/− 39% (42/107) α‐thalassemia 14% (10/74) α‐triplication 6% (10/165)	IVS‐I‐6 (T>C) (mild β^+^) 31% CD 39 (C>T) (β^0^) 24% IVS‐I‐110 (G>A) (β^+^) 17%	No transfusion‐47% (78/165) NTDT‐ 26% (42/165) TDT‐ 27% (45/165)	(Camaschella et al. 1995)
	Italy	23	91% (21/23) compound heterozygous or homozygous β‐thalassemia 9% (2/23) heterozygous (novel mutations)	Not systematically assessed (study focused on β‐globin mutation characterization); triple α‐globin gene reported in one family	IVS‐I‐6 (T>C) (mild β+) 26% (12/46 alleles) β‐101 (C>T) promoter 15% (7/46 alleles) CD 39 (β0) 15% (7/46 alleles) β‐87 (C>G) promoter (mild β+) 9% (4/46 alleles) 3 novel mutations (IVS‐I‐nt2 T>A; IVS‐II‐nt844 C>G; Cd126 ‐T) 7% (3/46 alleles)	Diagnosed as thalassemia intermedia by inclusion (implicit NTDT); overall transfusion‐status breakdown not reported for full cohort	(Murru et al. 1991)
	Greece	31	All are homozygous or compound heterozygous β‐TI	Mild β alleles (β^+^/β^+^or β^+^/β^0^) 72% (22/31) Xmn I Gγ+/+ & +/− 23% (7/31)	IVS‐I‐6 (T>C) (mild β^+^) 42% IVS‐I‐110 (G>A) (β^+^) 23% IVS‐I‐110 (G>A) (β^0^) 20%	All NTDT	(Kanavakis et al. 1995)
	Turkey	41	All are homozygous or compound heterozygous βTI	Mild β alleles (β^+^/β^+^or β^+^/β^0^) 51% (21/41) Xmn I Gγ +/+ 42% (17/41)	IVS‐I‐6 (T>C) (mild β^+^) 24% IVS‐II‐1 (G>A) (β^0^) 22% CD 8 (−AA) (β^0^) 20%	All NTDT	(C. Altay and Gürgey 1990)
	Israel	95 (60 families)	82% (49/60) Homozygous or compound heterozygous β‐TI 18% (11/60) Heterozygous β‐TI	Mild β alleles 62% (37/60) α triplication 5% (3/60)	IVS‐I‐6 (T>C) (mild β^+^) 40% −28 (A>C) (β^+^) 12% IVS‐II‐1 (G>A) (β^0^) 8% −101 (C>T) (β^++ silent^) 5%	Majority—NTDT	(Rund et al. 1997)
	Lebanon	73 (50 families)	All are homozygous or compound heterozygous β‐TI	Mild β alleles 68% (50/73) Xmn I Gγ +/+ 25% (18/73)	IVS‐I‐6 (T>C) (mild β^+^) 41% CD 29 (C>T) (mild β^+^) 12% IVS‐II‐1 (G>A) (β^0^) 9% −88 (C>T) (β^++^) 7%	No regular transfusion 30% (22/73) NTDT 59% (43/73) TDT 12% (9/73)	(Qatanani et al. 2000)
	Lebanon	21	67% (14/21) homozygous cd29 (C>T); 33% (7/21) compound heterozygous (cd29/IVS‐I‐1 or cd29/IVS‐II‐1)	BCL11A rs1427407/rs766432 effect allele 29% (6/21) HMIP rs28384513 effect allele‐ 24% (5/21) HBG2‐XmnI‐158 (C>T) 9.5% (2/21)	cd29 (C>T) homozygous 67% (14/21) cd29/IVS‐II‐1 (G>A) 19% (4/21) cd29/IVS‐I‐1 (G>A) 14% (3/21)	NTDT 57% (12/21) TDT 43% (9/21)	(Brancaleoni et al. 2019)
	Palestine	51	98% (50/51) Homozygous or compound heterozygous β‐TI 2% (1/51) Heterozygous β‐TI	Mild β alleles (β^+^/β^+^) 84% (43/51) Xmn I Gγ +/+ 6% (3/51) α thalassemia 2% (1/51) α triplication 2% (1/51)	IVS‐I‐6 (T>C) (mild β^+^) 74% IVS‐I‐110 (G>A) (β^+^) 8% IVS‐II‐1 (G>A) (β^0^) 7%	No regular transfusion 18% (9/51) NTDT 18% (9/51) TDT 65% (33/51)	(Faraon et al. 2019)
Europe	Spain	73	All 73 are heterozygous β‐TI thalassemia with α‐globin gene triplication/quadruplications	α‐globin gene triplication (ααα anti3.7) 100% (73/73) Co‐inherited ‐α3.7 deletion (1 case) 1.4% (1/73)	CD39 (C>T) (β0) 35.6% (26/73) IVS‐I‐nt1 (G>A) (β0) 23.3% (17/73) IVS‐I‐nt110 (G>A) (severe β+) 20.5% (15/73)	STI 24.7% (18/73) MTI 56.2% (41/73) TT 19.2% (14/73)	(Ropero et al. 2022)
	France	45	All 45 are heterozygous β‐thalassemia (β0 or β+) + triplication/quadruplication at HBA locus: heterozygous triplication 91% (41/45); homozygous triplication 9% (4/45)	ααα anti3.7 triplication (heterozygous) 91% (41/45) ααα anti3.7 triplication (homozygous, 6 functional α‐genes) 9% (4/45)	Heterozygous β0 or β + HBB mutation (specific alleles not detailed in source)	No transfusion 64.4% (29/45, heterozygous group) Occasional transfusion 29% (13/45) Transfusion‐dependent 7% (3/45, all homozygous triplication)	(Bonello‐Palot et al. 2023)
Middle East	Iraq	74 (51 families)	94% (48/51) Homozygous or compound heterozygous β‐TI 6% (3/51) Heterozygous β‐TI	Mild β alleles (β^+^/β^+^or β^+^/β^0^) 55% (28/51) Xmn I Gγ +/+ 22% (11/51) α thalassemia 8% (4/51)	IVS‐I‐6 (T>C) (mild β+) 33% IVS‐II‐1 (G>A) (β0) 21% CD 82/83 (−G) (β0) 10% CD 8 (−AA) (β0) 8%	No regular transfusion 69% (45/74) NTDT 31% (23/74) TDT 8% (6/74)	(Al‐Allawi et al. 2014)
	Iran	52	47 Homozygous or compound heterozygous β‐TI	Xmn I Gγ +/+ 47% (22/47) Mild β alleles 15% (7/47)	IVS‐II‐1 (G>A) (β0) 43% IVS‐I‐6 (T>C) (mild β+) 7% IVS‐I‐110 (G>A) (β+) 4%	No regular transfusion 21% (11/52) TDT 17% (9/52) (62% (32/52) received hydroxyurea)	(Neishabury et al. 2008)
	Iran	45	All are homozygous or compound heterozygous β‐TI	XmnI Gγ +/+ 62% (28/45) homozygous (TT) 27% (12/45)	IVS‐I‐5 (G>C) (44%) IVSI‐5 (G>C) /−88 (C>T) ‐(9%) IVS‐II‐1 (G>A)	NTDT; Variable phenotype	(Miri‐Moghaddam et al. 2017)
	Iran	38	Homozygous or compound heterozygous β‐thalassemia (38/39 identified); 1 additional case α‐globin deletion only (−α3.7/−MED, HbH disease)	Positive XmnI Gγ polymorphism 76.9% (60/78 chromosomes) α‐globin gene deletion (−α3.7 or ‐MED) 7.7% (6/78 chromosomes)	IVSII‐1 (G>A) (severe β+/β0) 56.4% (44/78) CD8 (−AA) 10.3% (8/78) IVS1‐6 (T>C) (mild β+) 6.4% (5/78)	Variable transfusion requirement; mean HbF > 50%; splenectomy in 53.8% (21/38)	(Arab et al. 2011)
	Iran (IVSII‐1 homozygotes)	42 TI vs. 61 TM	All homozygous IVSII‐1 (G>A)/IVSII‐1 (G>A); classified by clinical phenotype into thalassemia intermedia (*n* = 42) vs. thalassemia major (*n* = 61)	XmnI Gγ +/+ (TI vs. TM) 88.1% (37/42) vs. 70.5% (43/61), ns Co‐inherited α‐thalassemia (TI vs. TM) 19% (8/42) vs. 13.1% (8/61), ns	IVSII‐1 (G>A) homozygous 100% (103/103, by design)	TI 40.8% (42/103) TM—59.2% (61/103)	(Neishabury et al. 2010)
Asia	Pakistan	63	67% (42/63) Homozygous or compound heterozygous β‐TI; 14% (9/63) Heterozygous βTI	Mild β alleles (β^+^/β^+^or β^+^/β^0^) 40% (25/63) α thalassemia 21% (13/63) Xmn I Gγ +/+ 6% (4/63) α triplication 2% (1/63)	IVS‐I‐5 (G>C) (β^+severe^) 50% CD 8/9 (+G) (β^0^) 10% C 41/42 (–TCTT) (β^0^) 3.4%	Variable transfusion requirement	(Khan et al. 2015)
	India	73	68% (50/73) Homozygous or compound heterozygous β‐TI 32% (23/73) Heterozygous β‐TI	α thalassemia 23% (17/73) Xmn I Gγ +/+ 27% (20/73) Mild β alleles (β^+^/β^+^) 12% (9/73) α triplication 20.5% (15/73)	IVSI‐1 (G>T) (β^0^) 34% IVS‐I‐5 (G>C) (β^+severe^) 29% CD 41/42 (‐TCTT) (β^0^) 8% CD 8/9 (+G) (β^0^) 7%	All NTDT	(Panigrahi et al. 2006)
	South China	117	83% (97/117) Homozygous or compound heterozygous β‐TI with other β‐globin defects 17% (20/117) Heterozygous β‐TI	Mild β alleles (β^+^/β^+^or β^+^/β^0^) 41% (49/117) β^+^or β^0^/ HPFH δβ 9% (11/117) α thalassemia 6% (7/117) α triplication 4% (5/117)	CD 41/42 (‐TCTT) (β^0^) 19.6% IVS‐II‐ 654 (C>T) (β^+severe^) 9.3% −28 (A>G) (β^+^) 20.1%	Variable phenotype	(Chen et al. 2010)
	Sri Lanka	50	34% (17/50) Homozygous or compound heterozygous β‐TI 66% (33/50) Heterozygous β‐TI	α triplication 56% (28/50) α thalassemia 18% (9/50) Mild β alleles 10% (5/50) β+/HPFH 3 4% (2/50) Xmn I Gγ +/+ 2% (1/50)	IVS‐I‐ 5 (G>C) (β^+Severe^) 40% IVSI 1 (G>A) (β^0^) 19% CD 30 (G>C) (β^0^) 6% CD 16 (− C) (β^0^) 6% CD41/42 (‐TTCT) (β^0^) 6% CD 15 (−T) (β^0^) 6%	No regular transfusion 14% (7/50) NTDT 66% (33/50) TDT 20% (10/50)	(Perera et al. 2019)

*Note:* Percentages represent the frequency of individual genetic modifiers reported in each study and are not mutually exclusive. Phenotypic classifications were harmonized where possible into transfusion‐dependent thalassemia (TDT), non‐transfusion‐dependent thalassemia (NTDT), or no regular transfusion requirement based on reported transfusion status (Table S5).

Abbreviations: β‐TI, β‐thalassemia intermedia; HbF, fetal haemoglobin; MTI, Mild Thalassemia Intermedia; NTDT, non‐transfusion‐dependent thalassemia; STI, Severe Thalassemia Intermedia; TDT, transfusion‐dependent thalassemia; TT, Thalassemic Trait; XmnI, XmnI Gγ polymorphism.

^a^ Mixed β‐thalassemia cohort; β‐thalassemia intermedia subset ≈16%.

### 3.2 Geographic Distribution and Regional Patterns

β‐thalassemia is widely distributed across the Mediterranean basin, parts of Africa, the Middle East, the Indian subcontinent, Southeast Asia, and the Pacific Islands, largely reflecting historical selective pressure from malaria (Taher et al. 2018; Weatherall and Clegg 2001).

Based on the included studies (*n* = 21), findings were categorized into four major geographic regions: Mediterranean, European, Middle Eastern, and Asian. These regions demonstrated distinct patterns in the relative contribution of β‐globin mutation severity, HbF‐inducing modifiers, and α‐globin gene dosage, consistent with established genotype–phenotype frameworks (Taher et al. 2018; Thein 2018) (Figure 3, Table 1).

**FIGURE 3:**
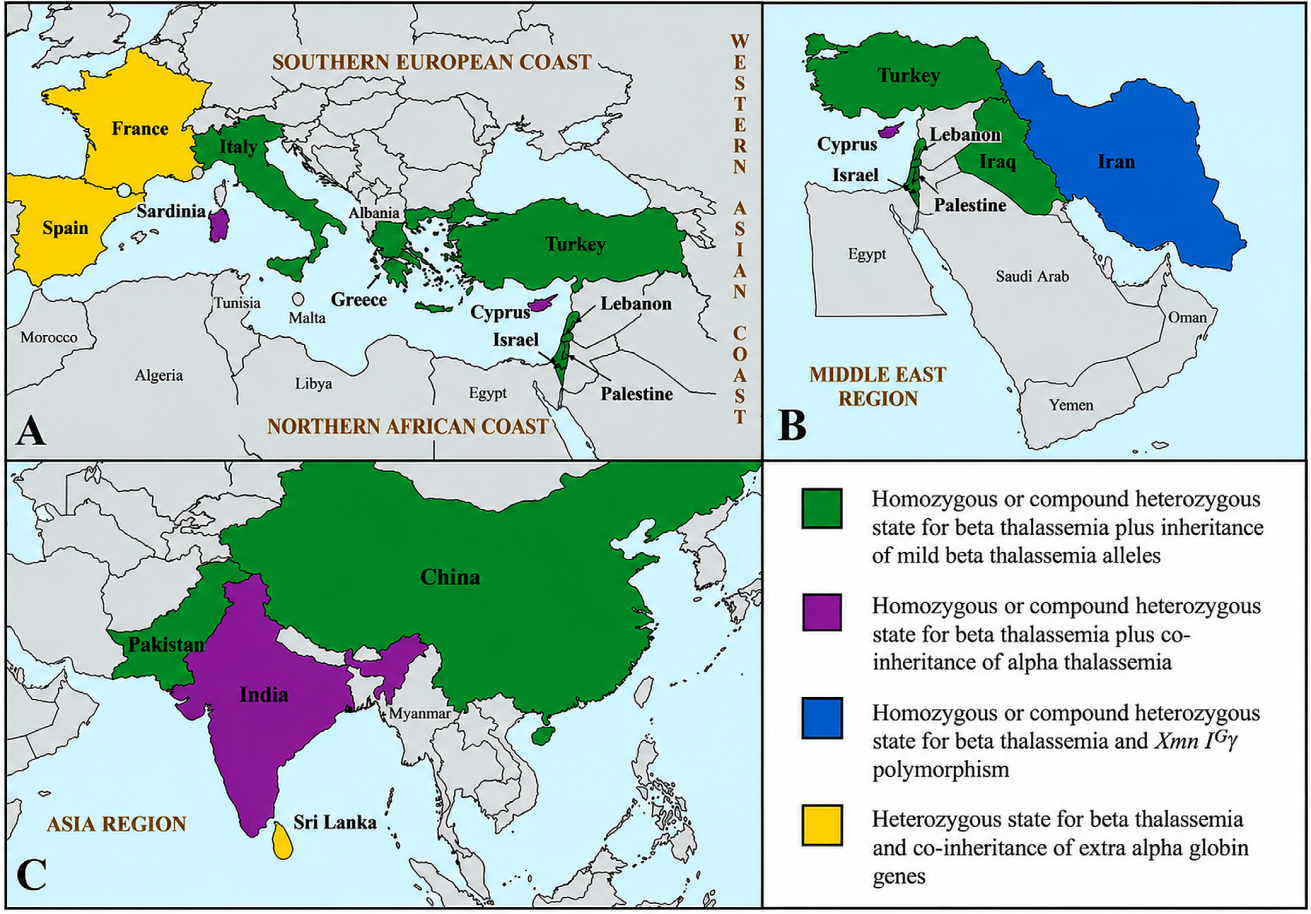
Geographic distribution of predominant genetic modifiers in β‐thalassemia intermedia. (A) Mediterranean and European region; (B) Middle East region; (C) Asian region. The colors indicate the primary modifying factor for each region.

#### 3.2.1 Mediterranean Region

As shown in Table 1, Mediterranean populations demonstrated a predominant pattern of β‐TI arising from homozygous or compound heterozygous states, accounting for approximately 82%–100% of cases. Mild β‐globin mutations were identified as the principal modifying factor in most cohorts (Camaschella et al. 1995; Kanavakis et al. 1995).

Studies from Cyprus and Sardinia highlight the significant role of α‐thalassemia co‐inheritance in modulating disease severity. In the Cypriot population, despite a high frequency of severe β‐globin mutations such as IVS‐I‐110 (G>A), accounting for approximately 74%–80% of alleles, co‐inheritance of α‐thalassemia was observed in about 52% of patients with β‐TI. This was associated with phenotypic amelioration, with patients demonstrating milder clinical courses and mean haemoglobin levels around 7.5 g/dL (Kountouris et al. 2016; Wainscoat et al. 1983).

Similarly, in Sardinia, despite the predominance of severe β^0^ mutations such as codon 39, all patients with thalassemia intermedia were non–transfusion‐dependent, with mean haemoglobin levels of approximately 9.7 g/dL. This phenotypic amelioration was strongly associated with co‐inherited α‐thalassemia, particularly the two‐gene deletion (−α/−α), as well as the presence of the XmnI polymorphism, which enhances fetal haemoglobin production (Galanello et al. 1989).

Studies from Italy, Turkey, and Greece consistently demonstrate that a substantial proportion of β‐TI cases are attributed to the presence of at least one mild β‐globin allele. In Italy, approximately 72% of patients did not require regular transfusions, and clinical presentation was often delayed, with diagnosis occurring after the age of 2 years. This is consistent with an earlier molecular characterization by Murru et al. (1991), who found that inheritance of at least one mild β‐thalassemia allele (β^+^‐IVS‐I‐6, β^−^87, or β^−^101), whether in the homozygous or compound heterozygous state, was the most common mechanism underlying the intermedia phenotype in 23 Italian patients; this study also identified three novel β‐thalassemia mutations (IVS‐I‐nt2 T>A, IVS‐II‐nt844 C>G, and a codon 126 single‐nucleotide deletion) in patients with atypical single‐allele genotypes, and, unlike other Italian cohorts, did not report a full cohort‐level transfusion‐status breakdown. Comparable findings were reported in Turkish and Greek cohorts, where most patients were non‐transfusion‐dependent (Ç. Altay and Gürgey 1990; Camaschella et al. 1995; Kanavakis et al. 1995; Murru et al. 1991).

Studies from the Eastern Mediterranean region demonstrate a broadly similar genetic framework to the wider Mediterranean population, predominantly driven by mild β‐globin mutations, particularly IVS‐I‐6 (T>C). However, considerable heterogeneity in clinical expression is observed across populations.

In Israeli cohorts, genetic heterogeneity was notable, with a proportion of cases remaining unexplained despite detailed molecular analysis (Rund et al. 1997). Lebanese patients frequently exhibited co‐inheritance of the XmnI Gγ polymorphism alongside mild β‐globin alleles, which was associated with increased HbF production and a predominantly non‐transfusion‐dependent phenotype (Qatanani et al. 2000).

In contrast, Palestinian cohorts, largely homozygous for IVS‐I‐6, demonstrated greater variability in Hb and HbF levels, with higher rates of transfusion dependence and splenectomy (Faraon et al. 2019). These findings highlight that while mild β‐globin mutations are the primary genetic basis in this region, HbF‐related modifiers play a critical role in determining phenotypic severity and contribute to inter‐population variability.

#### 3.2.2 European Region

A newly identified subset of European cohorts illustrates a distinct disease mechanism in which α‐globin gene dosage, rather than β‐globin mutation severity, is the primary driver of the β‐TI phenotype. In a Spanish cohort of 73 patients, β‐TI arose from the co‐inheritance of an α‐globin gene triplication (ααα‐anti3.7) with heterozygous β‐thalassemia; the great majority (94.5%, 69/73) carried a single extra α‐gene copy, while a smaller subset (6.8%, 5/73) also carried a co‐inherited δβ‐thalassemia or Hb Lepore allele. Despite this shared triplication‐driven mechanism, clinical severity varied considerably: 24.7% had a silent/mild phenotype, 56.2% a moderate phenotype, and 19.2% required regular transfusion (Ropero et al. 2022).

A similar mechanism was reported in a French cohort of 45 patients with heterozygous β‐thalassemia and co‐inherited α‐globin gene triplication or quadruplication. Most patients (91%, 41/45) carried a heterozygous triplication, while a smaller group (9%, 4/45) carried a homozygous triplication (six functional α‐genes). Transfusion requirements closely tracked triplication dosage: 64.4% of the heterozygous‐triplication group required no transfusion and 29% required occasional transfusion, whereas all patients requiring regular transfusion (7%, 3/45) carried the more severe homozygous triplication (Bonello‐Palot et al. 2023).

Together, these European cohorts demonstrate that α‐globin gene dosage alone, without a severe β‐globin mutation, can be sufficient to produce a β‐TI phenotype in an otherwise asymptomatic β‐thalassemia heterozygote, a mechanism distinct from the β‐globin‐ and HbF‐driven patterns observed in the Mediterranean and Middle Eastern regions, respectively.

#### 3.2.3 Middle Eastern Region

Across Middle Eastern cohorts, β‐TI is characterized by a distinct molecular profile in which HbF induction, most commonly mediated by the XmnI polymorphism, acts as the primary modifier of phenotypic expression (Thein 2018).

Data from Iranian cohorts clearly illustrate the dominant role of fetal haemoglobin–modulating mechanisms in determining the β‐thalassemia intermedia phenotype. In a molecular study by Maryam Neishabury et al., approximately 55% of patients carried the XmnI polymorphism in association with severe β‐globin mutations, resulting in significantly elevated HbF levels, with mean values around 56% (Neishabury et al. 2008). Similarly, a more recent cohort from South‐East Iran by Ebrahim Miri‐Moghaddam et al. demonstrated an even higher prevalence of XmnI polymorphism (~62%), with a strong association with markedly increased HbF levels, often exceeding 50% and reaching up to 85% in homozygous individuals (Miri‐Moghaddam et al. 2017). Together, these findings underscore the central role of HbF induction as the primary mechanism of phenotypic amelioration in this population. This mechanism also has important therapeutic implications, as patients with elevated HbF levels are more likely to respond favorably to HbF‐inducing agents such as hydroxyurea.

A similar, though less pronounced, pattern was observed in Iraqi cohorts, where XmnI polymorphism was present in 22% of cases alongside mild β‐globin alleles in 55%. This combination was associated with predominantly non‐transfusion‐dependent phenotypes (~72%) and moderate haemoglobin levels (mean 8.3 g/dL) (Al‐Allawi et al. 2014).

Collectively, these findings indicate that, in contrast to Mediterranean populations where β‐globin genotype predominates, phenotypic modulation in the Middle East is largely driven by HbF‐inducing mechanisms. Enhanced γ‐globin expression therefore represents the primary compensatory pathway mitigating β‐globin deficiency in this region.

#### 3.2.4 Asian Region

Across Asian cohorts, β‐TI demonstrate the greatest molecular heterogeneity, with a higher prevalence of heterozygous states and a dominant role for α‐globin gene modifications in modulating phenotype (Thein 2018).

In India, substantial genetic diversity is evident. Approximately 32% of β‐TI cases occur in heterozygotes. Among homozygous or compound heterozygous patients, α‐thalassemia co‐inheritance (23%) and XmnI polymorphism (22%) are key modifiers, whereas heterozygous cases are frequently associated with α‐globin gene triplication (19%) (Panigrahi et al. 2006). This distribution highlights the combined influence of globin chain imbalance and HbF‐inducing mechanisms in determining disease expression.

In Southern China, disease expression is more strongly driven by β‐globin genotype. Approximately 42% of patients carry at least one mild β‐allele, while α‐thalassemia co‐inheritance is relatively uncommon (~6%). The mutational spectrum is dominated by recurrent variants, particularly CD41/42 (‐TCTT) (36%) and IVS‐II‐654 (C>T) (25%), indicating a more genotype‐centric pattern of phenotypic modulation (Chen et al. 2010).

Sri Lanka demonstrates a distinct model in which heterozygosity plays a central role. Approximately 66% of cases occur in heterozygous individuals, with α‐globin gene triplication present in 56%, among the highest frequencies reported globally. This configuration exacerbates globin chain imbalance and converts otherwise asymptomatic carriers into clinically significant β‐TI, underscoring the critical role of α‐globin gene dosage in phenotypic determination (Perera et al. 2019).

### 3.3 Comparative Regional Synthesis

Comparative analysis across regions demonstrates distinct patterns in the relative contribution of genetic modifiers, summarized qualitatively in Figure 4. Mild β‐globin alleles were classified as the predominant modifier in Mediterranean populations, consistent with their frequent report across Italian, Greek, Turkish, Lebanese and Palestinian cohorts. HbF‐inducing polymorphisms, particularly the XmnI variant, were more prominent in Middle Eastern cohorts driven principally by the Iranian studies. α‐globin co‐inheritance, including gene triplication, was classified as predominant both in the European cohorts (France, Spain), where it was near‐universal, and in Asian populations, where it was consistently reported alongside dominance of different modifying mechanisms and underscore the heterogeneity of genotype–phenotype relationships (Figure 4).

**FIGURE 4:**
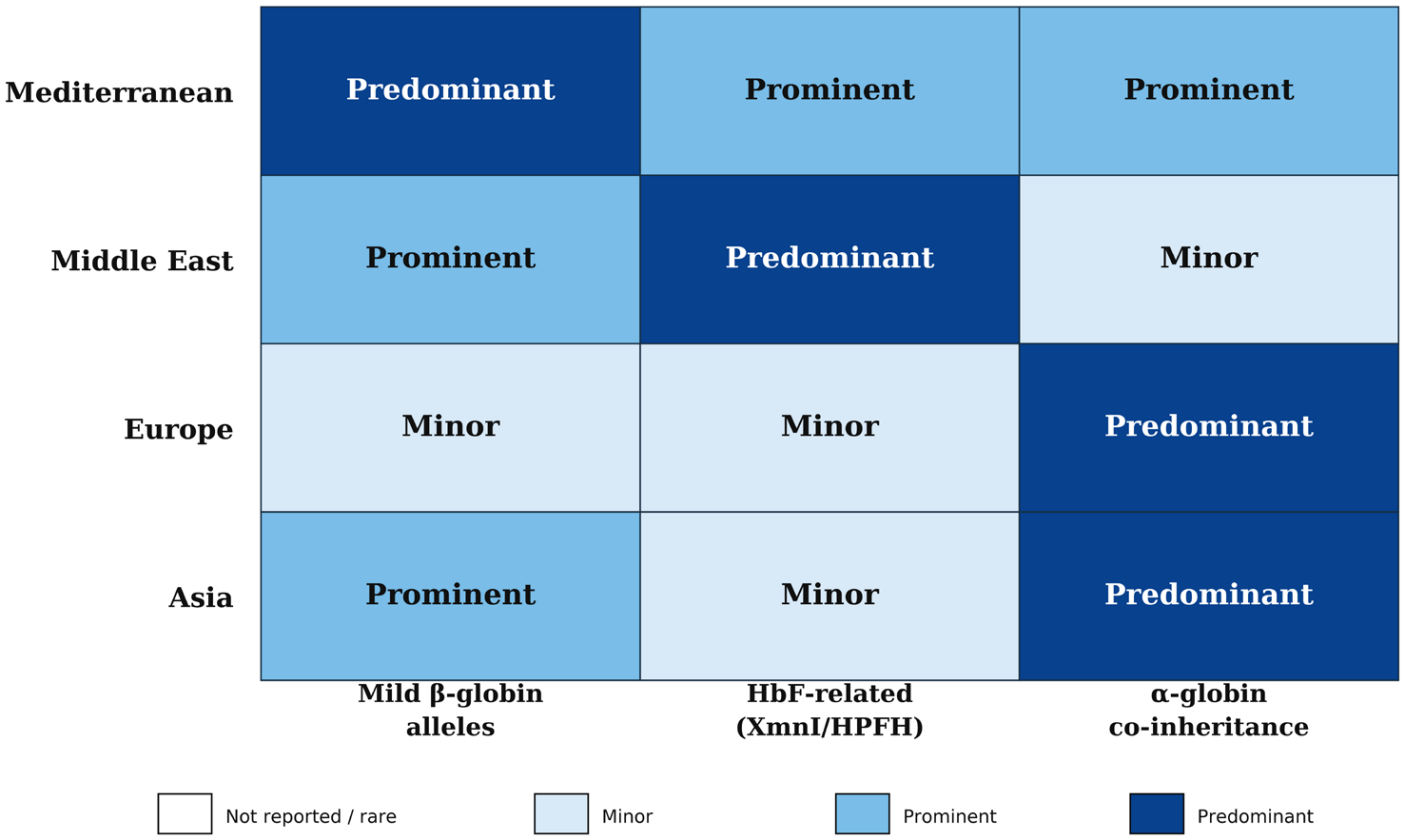
Regional distribution of dominant genetic modifiers in β‐thalassemia intermedia (qualitative evidence map). Qualitative evidence map showing the relative contribution of key genetic modifiers (mild β‐globin alleles, HbF‐related polymorphisms, α‐globin co‐inheritance) across four geographic regions.

Importantly, several studies reported patients in whom clinical phenotype could not be fully explained by known genetic modifiers, suggesting the presence of additional, yet unidentified regulatory factors contributing to disease variability.

## 4 Discussion

This systematic review suggests that the clinical heterogeneity of β‐TI observed among the included studies is not solely determined by β‐globin mutations but may also be substantially influenced by region‐specific genetic modifiers (Thein 2005, 2018). Distinct geographic patterns emerged among the included cohorts, reflecting differences in the relative contribution of β‐globin mutation severity, Hb‐F‐inducing polymorphisms, and α‐globin gene dosage; given the observational, heterogeneous, and geographically uneven nature of the included evidence, these patterns should be interpreted as trends within the available literature rather than established population‐level characteristics.

Comparative analysis across regions highlights clear differences in the relative contribution of genetic determinants. Among the included studies, Mediterranean cohorts were primarily influenced by the presence of mild β‐globin alleles, which account for a substantial proportion of cases and were associated with predominantly non–transfusion‐dependent phenotypes (Cao and Galanello 2010; Taher et al. 2018). In contrast, the included studies suggest that Middle Eastern populations tend to exhibit a higher prevalence of HbF‐inducing polymorphisms, particularly the XmnI variant, which contributes to phenotypic amelioration even in the presence of severe β‐globin mutations (Taher et al. 2018; Thein 2018). Among the included studies, European cohorts (Spain, France) and Asian populations both showed a dominant role for α‐globin gene alterations, including triplication, particularly among heterozygous individuals, where they significantly exacerbate globin chain imbalance (Mettananda 2015; Thein 2005). These findings suggest region‐specific differences in the relative contribution of genetic modifiers among the studies reviewed; however, these observations should be interpreted cautiously given the limited number of studies per region and substantial methodological heterogeneity and should not be generalized as established characteristics of entire populations.

Beyond their geographic distribution, the major genetic modifiers also differ in the magnitude and direction of their phenotypic effects. Mild β‐globin mutations generally exert one of the most substantial ameliorating influences by directly increasing residual β‐globin production, thereby reducing globin chain imbalance. HbF‐inducing modifiers, including the XmnI polymorphism and other regulatory loci, also substantially ameliorate disease severity by increasing γ‐globin expression and partially compensating for β‐globin deficiency, particularly in patients carrying severe β‐globin mutations. In contrast, co‐inheritance of α‐thalassemia generally has a moderate ameliorating effect through reduction of excess α‐globin chains, whereas α‐globin gene triplication is among the strongest disease‐exacerbating modifiers by increasing globin chain imbalance, particularly in heterozygous β‐thalassemia. Although the magnitude of these effects varies among individuals and populations, their overall direction of phenotypic modification was consistent across the included studies.

The central mechanism underlying phenotypic variability in β‐TI is globin chain imbalance, resulting from excess unpaired α‐globin chains (Thein 2005; Weatherall 2012). In Mediterranean populations, the presence of mild β‐globin mutations reduces this imbalance, resulting in milder disease. In contrast, Middle Eastern populations compensate through increased γ‐globin expression and elevated HbF levels, which partially offset β‐globin deficiency (Taher et al. 2018). In Asian populations, particularly in South Asia, excess α‐globin production due to gene triplication amplifies chain imbalance and leads to clinically significant disease even in heterozygous carriers (Mettananda 2015). These contrasting mechanisms likely reflect evolutionary adaptation to malaria, which has shaped the distribution of both β‐globin mutations and their modifiers across populations (Weatherall and Clegg 2001).

Beyond classical modifiers, emerging evidence highlights the importance of additional genetic regulators of HbF production. Genome‐wide association studies (GWAS) have identified key loci, including BCL11A and the HBS1L‐MYB intergenic region, as major determinants of HbF variability (Lettre et al. 2008; Sankaran et al. 2008). BCL11A acts as a transcriptional repressor of γ‐globin expression, whereas the HBS1L‐MYB locus influences erythropoiesis and HbF levels (Thein 2018). In addition, epigenetic mechanisms such as chromatin remodeling and DNA methylation further modulate globin gene expression (Bauer et al. 2012). These findings suggest that the genetic architecture of β‐TI extends beyond classical modifiers and involves a complex network of regulatory pathways that influence disease severity.

The geographic variability in molecular mechanisms has important clinical implications. First, it highlights the limitations of a uniform diagnostic approach. Population‐specific genetic testing strategies may improve diagnostic accuracy, such as prioritising α‐globin gene copy number analysis in Asian populations and HbF‐related polymorphisms in Middle Eastern cohorts (Taher et al. 2018). Second, the observed‐phenotype relationships may help inform future precision medicine approaches by identifying molecular pathways that could influence therapeutic decision‐making. Although therapies that increase HbF production, such as hydroxyurea and agents targeting ineffective erythropoiesis, such as luspatercept (Cappellini et al. 2020; Sankaran et al. 2008), may have differential effects according to the underlying molecular profile, the included studies did not directly evaluate treatment response. Therefore, these potential therapeutic implications should be considered hypothesis‐generating and require validation in prospective clinical studies.

To integrate these findings, a conceptual genotype–phenotype framework can be proposed in which β‐TI severity is determined by three interacting axes: (1) β‐globin mutation severity, (2) α‐globin gene dosage, and (3) HbF‐modulating factors (Figure 5). The relative contribution of these axes varies across geographic regions represented in the included studies, with β‐globin mutation severity more frequently reported as the dominant modifier among Mediterranean cohorts, HbF‐related induction among Middle Eastern (particularly Iranian) cohorts, and α‐globin gene dosage among Asian and the newly identified European cohorts. This framework is hypothesis‐generating rather than predictive or validated, is based on a limited and heterogeneous set of observational studies per region, and would require prospective validation before it could inform individualized management.

**FIGURE 5:**
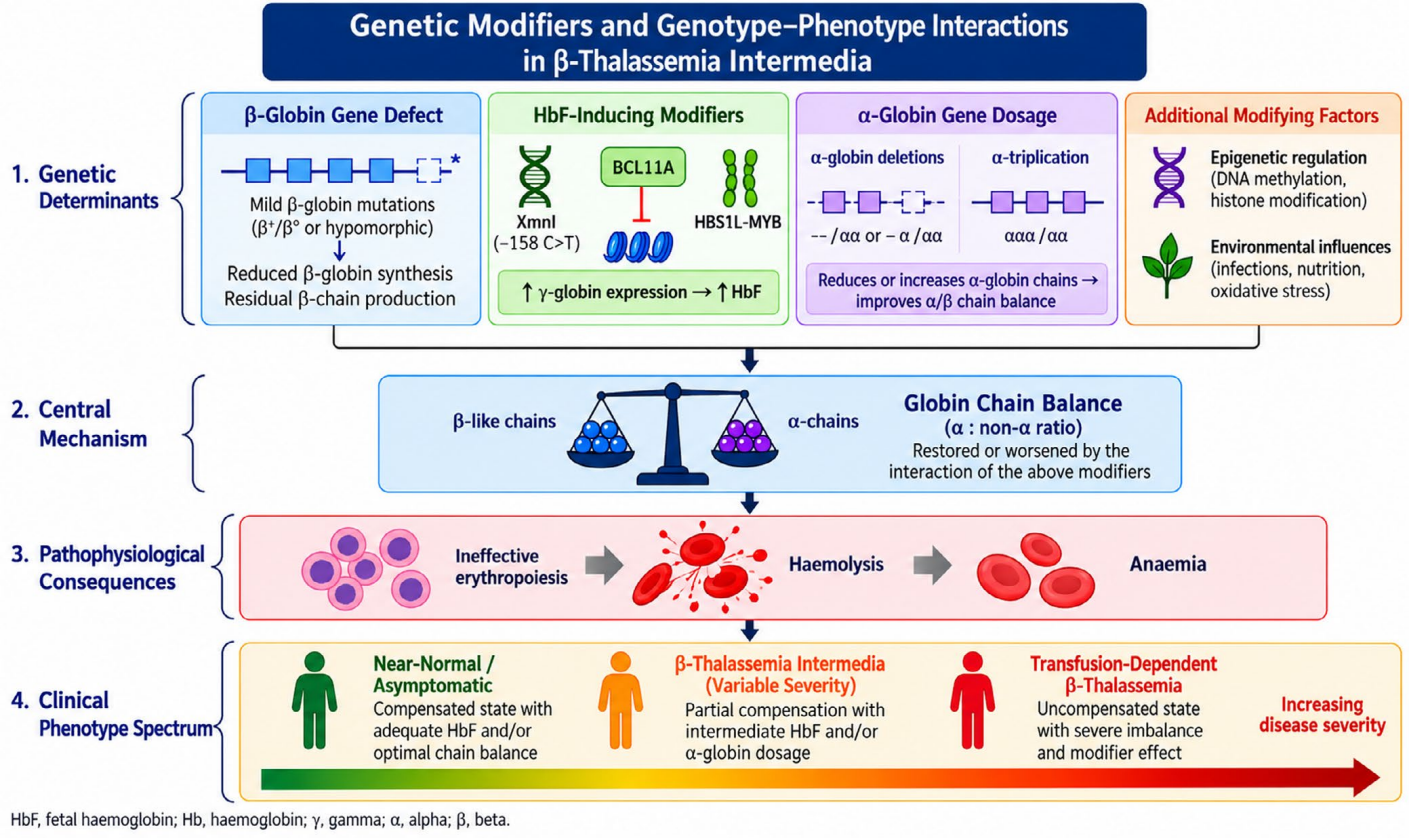
Mechanistic framework of genotype–phenotype interactions in β‐thalassemia intermedia. Disease severity reflects the combined effects of β‐globin mutation severity, HbF‐inducing genetic modifiers, and α‐globin gene dosage. These determinants, together with epigenetic and environmental modifiers, influence globin chain balance and thereby modulate ineffective erythropoiesis, haemolysis, and anaemia. The cumulative effect determines the clinical spectrum, ranging from compensated or asymptomatic disease to β‐thalassemia intermedia and transfusion‐dependent β‐thalassemia. Variation in the prevalence and interaction of these modifiers contributes to the geographical heterogeneity of disease phenotype.

This review focused specifically on non–HbE β‐thalassemia intermedia to enable clearer evaluation of classical genotype–phenotype modifiers without the additional complexity introduced by structurally abnormal haemoglobin variants. HbE β‐thalassemia, although highly prevalent in Southeast Asia, represents a distinct molecular entity with unique clinical and genetic characteristics (Cao and Galanello 2010). Inclusion of HbE‐associated disease would introduce additional heterogeneity and limit comparability across regions. Nevertheless, given its clinical importance, dedicated systematic evaluation of HbE β‐thalassemia remains an important area for future research.

Several limitations should be acknowledged. Most included studies were based on relatively small, single‐center cohorts, raising the possibility of selection bias. Therefore, the observed regional patterns should be interpreted as trends rather than definitive population characteristics. These methodological differences may have contributed to the observed between‐study heterogeneity and should be considered when interpreting regional patterns. In addition, variability in phenotypic classification, including inconsistent use of terms such as “mild,” “moderate,” “transfusion‐dependent thalassemia (TDT),” and “non–transfusion‐dependent thalassemia (NTDT),” limits comparability across studies and highlights the need for standardized definitions (Taher et al. 2018). Furthermore, the search strategy was limited to selected databases due to access constraints, which may have resulted in the omission of some relevant studies despite the use of supplementary search methods.

In conclusion, β‐thalassemia intermedia represents a genetically and clinically heterogeneous disorder shaped by region‐specific molecular mechanisms. Recognition of these patterns is essential for accurate diagnosis, prognostication, and individualized management. Future research integrating genomic data with standardized clinical phenotyping across diverse populations will be critical to advancing precision medicine in β‐TI.

## Author Contributions

**Kavindya Fernando:** investigation, formal analysis, writing – original draft, writing – review and editing. **Shiromi Perera:** conceptualization, methodology, formal analysis, investigation, writing – original draft, writing – review and editing. **Anuja Premawardhena:** validation, supervision, writing – review and editing.

## Funding

The authors have nothing to report.

## Disclosure

Transparency: Prof. Shiromi Perera affirms that this manuscript is an honest, accurate, and transparent account of the study being reported; that no important aspects of the study have been omitted; and that any discrepancies from the study as planned have been explained.

Registration and Protocol: This systematic review was conducted in accordance with the PRISMA 2020 guidelines. The protocol was not prospectively registered with PROSPERO. The authors acknowledge this as a limitation.

## Ethics Statement

Ethical approval was not required for this study as it is a systematic review based entirely on previously published data. No primary data were collected, and no human participants were directly involved in this research.

## Conflicts of Interest

The authors declare that the research was conducted in the absence of any commercial or financial relationships that could be construed as a potential conflicts of interest.

## Supporting information


**Table S1:** Complete PubMed Search Strategy.

**Table S2:** Full‐Text Screening and Eligibility Assessment Log.

**Table S3:** Data Extraction Log of Included Studies.

**Table S4:** Quality Assessment of Included Studies Using an Adapted Newcastle–Ottawa Scale.

**Table S5:** Phenotype Harmonization Crosswalk.

## Data Availability

The data that supports the findings of this study are available in the Supporting Information of this article.
