# Supplementary material for: Genotypic‐Phenotypic Correlations in Non‐HbE β‐Thalassemia Intermedia: A Geographic Systematic Review
Source: Mol Genet Genomic Med. 2026 Nov 2;14(11):e70330. doi: 10.1002/mgg3.70330 (PMC13728551; doi:10.1002/mgg3.70330)
Supplement: Supplementary file 1 [file MGG3-14-e70330-s001.docx]

**Supplementary Table S4. Quality Assessment of Included Studies (Adapted Newcastle–Ottawa Scale)**

| **No.** | **Study** | **Country** | **Selection** | **Comparability** | **Outcome** | **Overall Quality** | **Key Justification** |
| --- | --- | --- | --- | --- | --- | --- | --- |
| 1 | Wainscoat et al., 1983 | Cyprus | ★★★ | ★★★ | ★★★ | High | Clear cohort; α-thalassemia effect well demonstrated with significance testing (P<0.01); note this study characterizes only α-globin genotype -- no specific β-globin mutations are identified or named for any patient |
| 2 | Galanello et al., 1989 | Sardinia | ★★★ | ★★★ | ★★★ | High | Well-defined TI vs TM; detailed β, α and XmnI analysis |
| 3 | Camaschella et al., 1995 | Italy | ★★★ | ★★★ | ★★★ | High | Large cohort; extensive genotype–phenotype comparison |
| 4 | Murru et al., 1991 | Italy | ★★ | ★★ | ★★★ | Moderate | Small sample; no modifier analysis; strong mutation characterization |
| 5 | Kanavakis et al., 1995 | Greece | ★★★ | ★★ | ★★★ | Moderate–High | Clear cohort; β mutations + XmnI studied; however α-thalassemia was not assessed at all in this paper, leaving one of the three classic modifiers unexamined |
| 6 | Altay & Gürgey, 1990 | Turkey | ★★★ | ★★ | ★★★ | Moderate–High | Good mutation data; limited statistical comparison |
| 7 | Rund et al., 1997 | Israel | ★★★ | ★★★ | ★★★ | High | Large cohort; strong heterogeneity analysis |
| 8 | Qatanani et al., 2000 | Lebanon | ★★★ | ★★ | ★★★ | Moderate–High | Good molecular data; phenotype confounded by treatment history |
| 9 | Faraon et al., 2019 | Palestine | ★★★ | ★★★ | ★★★ | High | Modern multicentre study; robust molecular and clinical data |
| 10 | Al-Allawi et al., 2014 | Iraq | ★★★ | ★★★ | ★★★ | High | XmnI and α-thalassemia genotyping; extensive clinical complications documented (pulmonary hypertension, iron overload, viral hepatitis screening). Note: BCL11A/HBS1L-MYB were NOT studied in this paper -- that QTL panel belongs to a different Al-Allawi study (2015, 102 Baghdad patients), not this one (74 patients, Northern Iraq). |
| 11 | Neishabury et al., 2008 | Iran | ★★ | ★★★ | ★★★ | Moderate–High | Strong mechanistic classification; mixed cohort definition |
| 12 | Miri-Moghaddam et al., 2017 | Iran | ★★★ | ★★ | ★★★ | Moderate–High | Strong XmnI–HbF association; limited modifier scope |
| 13 | Khan et al., 2015 | Pakistan | ★★★ | ★★ | ★★★ | Moderate–High | Multi-ethnic cohort; limited phenotype stratification |
| 14 | Panigrahi et al., 2006 | India | ★★★ | ★★★ | ★★★ | High | Strong analysis of α-triplication and multiple modifiers |
| 15 | Chen et al., 2010 | China | ★★★ | ★★★ | ★★★ | High | Large cohort; extensive modifier analysis including advanced genes |
| 16 | Perera et al., 2019 | Sri Lanka | ★★★ | ★★★ | ★★★ | High | Multicentre study; strong α-globin mechanism and phenotype correlation |
| 17 | Neishabury et al., 2010 | Iran | ★★★ | ★★ | ★★★ | Moderate–High | Controlled design isolating a single primary mutation background (IVSII-1 homozygotes only, n=103: 42 TI vs 61 TM) to test modifier effects; found no significant difference in XmnI Gγ+/+ (88.1% vs 70.5%) or coinherited α-thalassemia (19% vs 13.1%) between groups, highlighting the limits of these two modifiers alone. Predates BCL11A/HBS1L-MYB genotyping. |
| 18 | Arab et al., 2011 | Iran | ★★★ | ★★★ | ★★ | Moderate–High | Reasonably sized cohort (n=38) with a comprehensive modifier panel: β-globin mutation, α-globin deletion, XmnI polymorphism, and full β-globin cluster RFLP haplotype analysis (7 sites). However, comparisons are largely descriptive with no formal statistical significance testing between subgroups. |
| 19 | Brancaleoni et al., 2018 | Lebanon | ★★ | ★★★ | ★★★ | Moderate–High | Focused, well-characterized cohort restricted to a single β-globin mutation (cd29, n=21) with thorough genotyping of 10 HbF-modifier SNPs (BCL11A, HMIP, XmnI-158) and statistical testing (Kruskal-Wallis, Fisher's exact); however small sample size limits statistical power, and none of the modifier associations reached conventional significance despite favourable trends. |
| 20 | Ropero et al., 2022 | Spain | ★★★ | ★★ | ★★★ | Moderate–High | Largest published series (n=73) of β-thalassemia heterozygotes with co-inherited α-globin gene triplication/quadruplication (ααα-anti3.7), with clear genotype-based grouping and robust statistical comparison of hematological severity (Mann-Whitney/Kruskal-Wallis). However, the analysis is restricted to α-gene copy number as the sole modifier; other classic modifiers (XmnI, BCL11A, HBS1L-MYB) were not assessed. |
| 21 | Bonello-Palot et al., 2023 | France | ★★★ | ★★★ | ★★★ | High | National multicentre registry study (NaThalY, n=45 TIA vs n=160 TIB control cohort) with robust statistical comparison (Chi-2, Fisher's exact, Student's t-test/Mann-Whitney) across transfusion needs, iron overload (ferritin and hepatic/cardiac MRI), and complication rates (cholelithiasis, splenectomy, extramedullary haematopoiesis). Does not examine additional HbF modifiers (XmnI, BCL11A) beyond α-globin gene triplication status. |

Selection: Assessment of cohort definition, sample size, and inclusion criteria for β-thalassemia intermedia.
Comparability: Evaluation of genetic modifiers and genotype–phenotype relationships.
Outcome: Quality of clinical and molecular data reporting, including transfusion status and severity.
Overall Quality:

- High: robust methodology with comprehensive molecular and clinical analysis
- Moderate–High: minor limitations (e.g., incomplete modifier analysis)
- Moderate: limited sample size or incomplete genetic evaluation
