# Supplementary material for: Genotypic‐Phenotypic Correlations in Non‐HbE β‐Thalassemia Intermedia: A Geographic Systematic Review
Source: Mol Genet Genomic Med. 2026 Nov 2;14(11):e70330. doi: 10.1002/mgg3.70330 (PMC13728551; doi:10.1002/mgg3.70330)
Supplement: Supplementary file 2 [file MGG3-14-e70330-s003.docx]

## **Supplementary Table S5. Phenotype Harmonization Crosswalk**

| Country | Study | Original criteria / terminology as reported | Harmonized category | Harmonization notes |
| --- | --- | --- | --- | --- |
| Cyprus | Wainscoat et al., 1983 | NTDT 81% (22/27); TDT 18% (5/27) | NTDT / TDT | Clean binary split as reported. |
| Sardinia | Galanello et al., 1989 | "All non–transfusion-dependent" | NTDT (all) | Cohort defined by non-transfusion-dependence at recruitment. |
| Italy | Camaschella et al., 1995 | No transfusion 47% (78/165); NTDT 26% (42/165); TDT 27% (45/165) | 3-tier as reported | Only study in this set using all three tiers with a full cohort breakdown. |
| Italy | Murru et al., 1991 | No cohort-level transfusion-status breakdown reported; diagnosis of "thalassemia intermedia" was itself the inclusion criterion | NTDT (implicit, by inclusion) | Cannot be placed on the 3-tier scale with real data; harmonization here is an assumption based on diagnostic label, not a reported statistic. |
| Greece | Kanavakis et al., 1995 | "All NTDT" | NTDT (all) | By inclusion design. |
| Turkey | Altay & Gürgey, 1990 | "All NTDT" | NTDT (all) | By inclusion design. |
| Israel | Rund et al., 1997 | "Majority – NTDT"; exact proportions not tabulated | NTDT (majority, unquantified) | Original paper does not give a precise transfusion breakdown. |
| Lebanon | Qatanani et al., 2000 | No regular transfusion 30% (22/73); NTDT 59% (43/73); TDT 12% (9/73) | 3-tier as reported |  |
| Lebanon (cd29 subgroup) | Brancaleoni et al., 2018 | NTD 57% (12/21); TD 43% (9/21) | NTDT / TDT | Binary classification; no intermediate "occasional transfusion" tier used. |
| Palestine | Faraon et al., 2019 | No regular transfusion 18% (9/51); NTDT 18% (9/51); TDT 65% (33/51) | 3-tier as reported |  |
| Spain | Ropero et al., 2022 | "STI" (silent/mild TI) 24.7% (18/73); "MTI" (moderate TI) 56.2% (41/73); "TT" (transfusion-dependent thalassemia) 19.2% (14/73) | STI→NTDT (no/rare transfusion); MTI→NTDT (occasional transfusion); TT→TDT | Study uses its own severity-tier nomenclature (STI/MTI/TT); mapped onto our 3-tier scheme by transfusion frequency, not severity label. |
| France | Bonello-Palot et al., 2023 | No transfusion 64.4% (29/45); Occasional transfusion 29% (13/45); Transfusion-dependent 7% (3/45) | 3-tier as reported |  |
| Iraq | Al-Allawi et al., 2014 | Never transfused 32.4% (24/74); of the remaining 50 transfused patients, 83.8% had ≤3 transfusions/year and 16.2% had >3/year | No regular transfusion 32% (24/74); NTDT (≤3/yr) 57% (42/74); TDT (>3/yr) 11% (8/74) | Original Table 1 draft had mis-based the 83.8%/16.2% subgroup percentages against the full cohort (n=74) rather than the 50 transfused patients, producing fractions (62/74, 12/74) not found in the source paper. Corrected here and in Table 1 by rebasing to the full cohort: 42/74 and 8/74. |
| Iran | Neishabury et al., 2008 | No regular transfusion 21% (11/52); TDT 17% (9/52); remainder (62%) not explicitly labelled | No regular transfusion / [unlabelled] / TDT | Original paper does not name the remaining 62%; likely NTDT by exclusion, but this is our inference, not a reported category — flagged accordingly. |
| Iran | Miri-Moghaddam et al., 2017 | “NTDT; variable phenotype” – no numerical breakdown | NTDT (all, unquantified) |  |
| Iran | Arab et al., 2011 | “Variable transfusion requirement”; splenectomy in 53.8% (21/38); no transfusion-status breakdown given | Not classifiable on 3-tier scale | Reports splenectomy rate and mean HbF instead of transfusion status; cannot be harmonized without assumption. Left unclassified rather than forced. |
| Iran | Neishabury et al., 2010 | Study’s own classification: TI 40.8% (42/103) vs TM 59.2% (61/103) | TI→NTDT; TM→TDT | Study explicitly compares TI vs TM as its primary design, using clinical diagnosis rather than transfusion frequency; mapped by definitional equivalence, not a reported frequency count. |
| Pakistan | Khan et al., 2015 | “Variable transfusion requirement” – no numerical breakdown | Not classifiable on 3-tier scale |  |
| India | Panigrahi et al., 2006 | Inclusion required Hb 7–9 g/dL with ≤4 transfusions/year or none | NTDT (all, by inclusion design) | Transfusion independence was an eligibility criterion, not an observed outcome distribution. |
| South China | Chen et al., 2010 | “Variable phenotype” – no numerical breakdown | Not classifiable on 3-tier scale |  |
| Sri Lanka | Perera et al., 2019 | No regular transfusion 14% (7/50); NTDT 66% (33/50); TDT 20% (10/50) | 3-tier as reported |  |
