# Supplementary material for: Genotypic‐Phenotypic Correlations in Non‐HbE β‐Thalassemia Intermedia: A Geographic Systematic Review
Source: Mol Genet Genomic Med. 2026 Nov 2;14(11):e70330. doi: 10.1002/mgg3.70330 (PMC13728551; doi:10.1002/mgg3.70330)
Supplement: Supplementary file 3 [file MGG3-14-e70330-s005.docx]

**Supplementary Table S1. Complete PubMed Search Strategy**

| **Item** | **Details** |
| --- | --- |
| **Database** | PubMed |
| **Search date** | 6 September 2026 |
| **Coverage** | Database inception to 6 September 2026 |
| **Language filter** | English |
| **Species filter** | Humans |
| **Search strategy** | (("beta-Thalassemia"[MeSH] OR "thalassemia intermedia" OR "beta thalassemia intermedia" OR "β-thalassemia intermedia")) AND ((genotype OR mutation OR polymorphism OR genetics OR "genetic modifier*" OR "alpha globin" OR "α-globin" OR "gamma globin" OR HbF OR "fetal haemoglobin")) AND ((phenotype OR "clinical severity" OR "clinical features" OR "disease severity" OR transfusion OR "transfusion dependence" OR "clinical outcome*")) |

**Additional Search Methods**

- **Google Scholar:** Search terms included *"beta thalassemia intermedia genotype phenotype"*, *"β-thalassemia intermedia genetic modifiers"*, and related combinations. The first 200 results were screened for potentially relevant studies.
- **Citation tracking:** Reference lists of included studies and relevant review articles were manually screened.
- **PubMed Similar Articles:** The "Similar Articles" feature was used to identify additional eligible publications.
